# The Use of Zolpidem in Catatonia: A Case Report

**DOI:** 10.7759/cureus.73493

**Published:** 2024-11-11

**Authors:** Aliu O Yakubu, Stuart Gibson, Rolinda Smit

**Affiliations:** 1 General Psychiatry, University Hospital Wishaw, Wishaw, GBR; 2 Old Age Psychiatry, University Hospital Wishaw, Wishaw, GBR

**Keywords:** cannabis induced catatonia, excited catatonia, malignant cata, past psychiatric history and depression, public psychiatry, subtypes of catatonia

## Abstract

Catatonia is a complex neuropsychiatric syndrome characterised by abnormal psychomotor disturbance, which poses a diagnostic and treatment challenge to clinicians. It is a life-threatening condition in its severe form, termed malignant and characterised by hyperthermia and autonomic disturbances. Early recognition and treatment are important in its management. A screening instrument, such as the Bush-Francis Catatonia Rating Scale (BFCRS), can help the clinician make a prompt diagnosis. Only a few published cases describe zolpidem being used to treat this condition, with lorazepam and electroconvulsive therapy (ECT) more commonly utilised as part of established treatment protocols. We discuss a case of a 70-year-old female with treatment-resistant depression and catatonic features who was successfully managed with zolpidem. This report highlights the role and efficacy of zolpidem in the clinical management of catatonia.

## Introduction

Catatonia is a neuropsychiatric syndrome marked by abnormal psychomotor disturbance (movements, behaviours, and withdrawal) often seen in a range of medical conditions and mental health disorders [1,2]. It is categorised into three subtypes: retarded, excited, and malignant (hypokinetic, hyperkinetic, and parakinetic). Hypokinetic catatonia is associated with immobility, negativism, and rigidity, among other symptoms, while hyperkinetic catatonia manifests as prolonged periods of psychomotor agitation, and parakinetic catatonia is characterised by abnormal movement. Many patients with catatonia present with a mixed symptom profile. Early recognition and treatment are important since dehydration and starvation, due to withdrawal, and immobility in catatonia could lead to life-threatening complications including malnutrition, infections, deep venous thromboses, pulmonary emboli, pressure ulcers, or muscle contractures [1].

The description of catatonia has been a subject of debate for years and early descriptions were provided by Dr. Karl Kahlbaum in the late 19th century, who recognised it as a disease entity [3]. However, it continued to be classified as a subtype of schizophrenia in several editions of the International Classification of Diseases (ICD) and the Diagnostic and Statistical Manual of Mental Disorders (DSM). With the emergence of new evidence and research, it is now recognised as a separate disease entity and this change is reflected in the DSM-5 and ICD-11 [4,5]. The diagnostic criteria in DSM-5 for the diagnosis of catatonic syndrome require three or more of the following symptoms: stupor, waxy flexibility, catalepsy, mutism, posturing, negativism, stereotypes, mannerisms, grimacing, agitation, echopraxia, and echolalia [4]. The Bush-Francis Catatonia Rating Scale (BFCRS) is the most widely used validated catatonia rating scale for diagnosis and evaluating treatment response. It comprises 23 items, with features overlapping with the DSM-5 and ICD-11 criteria [6].

The prevalence of catatonia has been reported to be between 5 and 20% in the acute inpatient psychiatric setting [2,7]. While its pathophysiology is yet to be fully understood, imaging has shown right medial orbitofrontal and lateral orbitofrontal prefrontal cortex dysfunction. Several neurotransmitters such as GABA, glutamate, serotonin, and dopamine transmissions have been implicated in the initiation and progression of catatonia symptoms [2]. Medications modifying this pathway are used for the management of catatonic symptoms. While benzodiazepines and electroconvulsive therapy (ECT) are widely considered the first-line treatment, newer evidence reported in several case reports has shown that zolpidem is effective in the treatment and diagnosis of catatonia [1,6].

We present a case of catatonia in a female in her 70s that was successfully managed with zolpidem. This report highlights the challenges in managing treatment-resistant depression with catatonic features and the importance of a multidisciplinary approach in optimizing patient outcomes.

## Case presentation

Our patient was an elderly woman who had first come to the attention of mental health services about four years ago due to an episode of acute-onset depressive features, possibly triggered by work-related stress. Before this, she had no prior history of mental health disorders. Despite trying numerous antidepressants (sertraline, mirtazapine, venlafaxine, vortioxetine, trazodone, and escitalopram), antipsychotics (quetiapine and olanzapine), anxiolytics, and lithium, her depressive symptoms had remained persistent. She had been hospitalised two years ago for a depressive episode with psychotic features characterised by low mood, loss of interest in enjoyable activities, and a preoccupation with food that had led her to stop eating at home. She had also exhibited possible catatonic features, including mutism and unusual behaviours such as repeatedly running up and down the stairs or moving from sitting to kneeling positions. At that time, she had undergone 15 ECTs twice a week with no significant improvement. Vascular depression had been suspected due to treatment resistance and atypical illness trajectory. The Edinburgh Cognitive and Behavioural ALS Screen (ECAS) had also shown dysexecutive features in her cognition. Notably, neuroimaging studies had shown non-specific frontal atrophy on MRI and minor frontal perfusion reduction on SPECT scans. Minimal changes had been observed between the scans conducted two years apart.

Approximately two months prior to this admission, her family described a deteriorating presentation. These episodes had involved prolonged periods of screaming incoherently and pacing around in a circular fashion in her room. She had been withdrawn, and eating less with objective signs of depression. The patient had experienced suicidal thoughts but denied intent. She had been commenced on regular lorazepam, which had been titrated up to 2 mg two to three times daily, as there had been some emerging concerns about catatonic features, but the response had been limited. Lorazepam had been gradually tapered off and discontinued before her admission.

She was subsequently admitted to a psychiatric ward for older people for a period of assessment. On mental state examination, she was well-groomed with intense eye contact and exhibited signs of anxiety and depression. She was guarded and showed suspicious behaviour towards the staff. She had good insight into her illness and there were no thought/ perception abnormalities. Physical examination revealed no positive findings. However, her blood tests indicated elevated white cell count with neutrophilia and there were three positive urine cultures for E. coli during the admission. Hence, she was commenced on prophylactic trimethoprim after review with urology. Further assessments by neurology revealed no focal neurological features but they recommended additional investigations including HIV, Lyme, syphilis virology, autoimmune encephalitis screen, and micronutrient levels, which were all unremarkable. Table 1 shows the investigation results.

**Table 1:** Investigation results HDL: high-density lipoprotein; LDL: low-density lipoprotein

Variable	Patient value	Reference range
Thyroid-stimulating hormone	2.64	0.27–4.2 mU/L
Angiotensin-converting enzyme	<15	20–70 U/L
Magnesium	0.82	0.7–1.0 mmol/L
Calcium	2.48	2.2–2.6 g/L
Erythrocyte sedimentation rate	2	1–50 mm/hr
Folate	3.24	3.9–26.80 ng/mL
Vitamin B12	328	197–771 pg/mL
HbA1c	40	20–42 mmol/mol
Rheumatoid factor	<10	0–14 IU/mL
Vitamin/metal		
Vitamin A	1.5	1.0–3.0 umol/L
Red blood cell vitamin B1	529	275–675 ng TDP/g Hb
Vitamin B2/haemoglobin ratio	1.5	1–3.4 nmol PAD/g Hb
Vitamin B6/haemoglobin ratio	370	250–680 pmol PLP/g Hb
Vitamin C	16	15–90 umol/L
Vitamin E	29	15–45 umol/L
Vitamin K/triglycerides	0.23	0.2–2.2 mmol/L
Copper	13.7	12–25 umol/L
Zinc	8.2	10–18 umol/L
Manganese	93	70–280 umol/L
Urea, electrolyte, and creatinine		
Sodium	142	133–146 mmol/L
Potassium	3.7	3.5–5.3 mmol/l
Chloride	106	95–108 mmol/l
Bicarbonate	23	22–29 mmol/l
Urea	4.7	2.5–7.8 mmol/l
Creatinine	74	60–110 mmol/l
Lipid profile		
Cholesterol	5.4	0–5 mmol/l
Triglyceride	1.8	0–2.3 mmol/l
HDL cholesterol	1.57	0.9–1.8 mmol/l
LDL cholesterol	3	0–3 mmol/l
Cholesterol/HDL ratio	3.4	0–5 mmol/l
Liver function test		
Alkaline phosphatase	18	5–55 U/L
Alanine transaminase	78	30–130 U/L
Bilirubin	18	0–20 U/L
Albumin	43	35–50 U/L
Full Blood count		
Haemoglobin	155	115–165 g/L
Platelet	251	150–400 ×10^9^/L
White blood cell count	14	4.5 to 11.0 ×10^9^/L

During the early week of the admission, her distress and agitation escalated, prompting an attempt to leave the ward. She was soon deemed to lack the capacity to give consent to treatment and was detained under the Mental Health Act. She was mostly in her room offering no interaction, with minimal oral intake, though there were short-lived episodes where she presented as bright and would converse freely with staff. During episodes of psychomotor agitation, she was observed to be grinding her teeth (grimacing) and walking in a jerky staggered fashion. Occasionally she was heard wailing, visibly distressed, and would place herself on the floor. Sudden and dramatic clinical responses within minutes were noticed with zolpidem administration for managing these episodes of acute distress. The Antecedent-Behaviour-Consequence (ABC) chart was employed in other to understand the trends and patterns of these episodes. The chart showed that the symptoms were most prominent in the morning and were exacerbated by staff attempts to provide verbal reassurance to her.

During a well-documented episode, she started with loud vocalisations and agitated pacing around the room, accompanied by abnormal posturing and grimacing. Her behaviour included intermittently leaving her bed to pace in a circular fashion with a distinctive gait, alternating between walking on tip-toes and undulating her head and torso. Throughout the episode, she made continuously loud and repetitive moans without coherent speech. Her only verbal communication consisted of echolalia, repeating sentences from nursing staff attempting to reassure her. She also displayed stereotypic movements with the occasional lifting of her hips on the bed.

Further examination demonstrated that whilst her upper limb tone was normal, she resisted extension of her lower limbs and this was proportionate to the force applied by the examiner (gegenhalten). During the examination, she rose again, displaying wailing and pacing behaviours in the same manneristic fashion. Her BFCRS score was 17 points. It was positive for excitement, mutism, staring, posturing/cataplexy, grimacing, echolalia, stereotypy, mannerism, negativism, withdrawal, impulsivity, and gegenhalten. Following the administration of 10 mg of zolpidem, her BFCRS score decreased to 6 points after 30 minutes, scoring only for mutism, staring, negativism, and withdrawal. She appeared more relaxed following this medication and was able to participate in conversation. At this stage, she struggled to describe her experience in detail, though she had described a build-up of inner tension and some generalised abdominal discomfort, leading to a feeling of “release” during the episode.

She was started on regular zolpidem as she and her family opted against ECT due to past non-response and hesitancy regarding lorazepam re-prescription. Her dose was gradually increased to 10 mg four times daily. Subsequently, significant improvement was observed as she engaged in outings with her family, ward-based activities, and interaction with other patients. An improvement was noted in her ability to attend to her personal care and her oral intake increased. Venlafaxine was commenced to manage the depressive episode and was gradually titrated to 75 mg in the morning and 37.5 mg at night. Her progress remained stable, leading to her discharge after 10 weeks of inpatient admission. The patient has shown continued improvement and has not required readmission over the past six months. She remains on the same dosage of zolpidem, as previous attempts to reduce the dose had led to concerns about the recurrence of catatonic features.

## Discussion

Catatonia has always presented a diagnostic and treatment dilemma. Prior to 2022, there had been no standardised treatment guidelines for the management of catatonia in the United Kingdom [6]. To address this gap, the British Association for Psychopharmacology (BAP) convened a group of experts to review existing literature and evidence. This effort led to the development of a national guideline for managing catatonia, which includes zolpidem as one of the effective treatment options.

This report showed the efficacy of zolpidem in the management of catatonia in an elderly patient, offering new alternative treatment approaches to benzodiazepine and ECT, which are the first-line treatments. There are few case reports of catatonia responsive to zolpidem. Mastain et al. [8] reported a case of catatonia in a 56-year-old female with a subcortical stroke who was successfully treated after treatment resistance to benzodiazepines and ECT. It has since been demonstrated to be highly effective in cases where catatonia has been refractory to Lorazepam and ECT in bipolar affective disorder [9] and schizoaffective disorder [10]. Case studies have also demonstrated zolpidem's efficacy in treating catatonia associated with other medical disorders such as frontotemporal dementia [11], paraneoplastic encephalitis [12], and systemic lupus erythematosus [13]. Zolpidem is also useful in the clinical diagnosis of catatonia, in the form of the zolpidem challenge test [14].

Zolpidem is a non-benzodiazepine sedative-hypnotic agent and is a positive allosteric modulator of GABA-A receptors, which shows specific binding to the alpha-1 subunit [15]. The cortico-cortical circuit, involving the primary motor cortex (PMC), supplementary motor area (SMA), and medial prefrontal cortex, controls motor activity and speed [16]. Reduced GABA-A receptor concentration in this part of the brain has been associated with catatonia, leading to decreased inhibition and disorganised motor control. Zolpidem acts by increasing CNS inhibition via the GABA-A receptor-positive allosteric effect. Restoration of the inhibitory control has been hypothesised to be responsible for its observed efficacy in catatonia. Neuroimaging studies have shown evidence of increased activity in GABA-ergic signalling pathways [17].

The recommended daily dosage for zolpidem in catatonia is 7.5-40 mg [6,18]. The decision to initiate regular zolpidem therapy in our case was guided by the patient's history of inadequate response to ECT and her family's concerns about increased agitation following the discontinuation of lorazepam, which could be related to benzodiazepine withdrawal. Subsequent clinical improvements, including enhanced engagement in daily activities and social interactions, underscored the therapeutic efficacy of zolpidem in managing severe catatonia.

## Conclusions

Based on our findings, zolpidem offers a promising alternative in the management of catatonia when first-line interventions have failed or are unavailable. Further research should focus on dosing regimens, long-term efficacy, and safety considerations regarding the use of zolpidem in catatonia.
